# Correspondence Principle Approach to Time-Dependent Decaying Quantized Fields

**DOI:** 10.3390/e28101160

**Published:** 2026-10-21

**Authors:** Irán Ramos-Prieto, Nikolai Korneev, Héctor M. Moya-Cessa

**Affiliations:** Instituto Nacional de Astrofísica Óptica y Electrónica, Calle Luis Enrique Erro No. 1, Santa María Tonantzintla 72840, Puebla, Mexico; iran@inaoep.mx (I.R.-P.); korneev@inaoep.mx (N.K.)

**Keywords:** correspondence principle, lindblad operators, master equation, time-dependent fields

## Abstract

We present a correspondence principle framework that maps a classical field-amplitude decay law Γ(t) onto a vacuum-loss quantum master equation for a single bosonic mode. Under the strict assumption that an initially coherent state remains pure, the ansatz selects a vacuum reservoir loss model rather than fixing general open-system quantum dynamics. While classical attenuation alone does not fix the noise structure accordingly, the derived generator is a compatible quantum model under explicit zero-temperature assumptions, not a consequence of the classical envelope alone—as thermal noise or environmental fluctuations can produce different quantum states with identical mean amplitudes. After reviewing representative classical regimes we derive a generalized master equation for a single bosonic mode whose complex amplitude is αΓ(t), where Γ(t) is a real, positive, strictly monotonic envelope. The resulting dynamics reduces to the standard amplitude-damping Lindblad equation for Γ(t)=exp(−γt/2) and preserves the coherent-state form |αΓ(t)〉〈αΓ(t)|. We clarify the exact physical assumptions required for this construction and distinguish CP-divisibility from non-Markovian memory effects. We prove uniqueness of this generator only within the stated pure-loss, single-mode, real-envelope assumptions, clarify its exact equivalence to a time-reparametrized pure-loss channel and discuss the limits of validity in dispersive absorbing media within macroscopic QED.

## 1. Introduction

The decay of electromagnetic (EM) fields is a central theme in electrodynamics, with manifestations ranging from charge relaxation in conductors and magnetic diffusion to radiation damping and cavity losses [1,2,3]. Classically, the functional form of the decay, exponential, diffusive, power law, oscillatory damped, stretched exponential, or multi-rate is fixed by the material response, boundary conditions, and geometry [4]. In many applications the decay envelope is not merely a nuisance parameter, it is an experimentally accessible fingerprint of loss channels, transport mechanisms, and mode structure. A unified way of translating such classical decay laws into a quantum description is therefore useful both conceptually and as a modeling tool.

When the field is quantized, dissipation is most often modeled by coupling the mode of interest to a thermal reservoir of harmonic oscillators, including the T=0 limit [5,6,7,8]. Under the Markovian approximation this construction yields a Lindblad master equation with a constant damping rate γ, a structure that guarantees complete positivity and underpins a large fraction of textbook quantum-optical models. This widely used limit is, however, tightly linked to an exponentially decaying classical amplitude, Γ(t)∝exp(−γt/2), and it effectively builds the exponential envelope into the generator. As a result, describing decay laws that are time-dependent or non-exponential typically requires either a microscopic non-Markovian treatment or a phenomenological generalization whose physical interpretation and consistency must be checked.

Many realistic systems deviate strongly from a single exponential. Disordered and complex dielectrics often display stretched exponential Kohlrausch–Williams–Watts relaxation [9,10], open resonators and nanophotonic structures can exhibit multi-mode quasi-normal-mode decay with several competing rates [11,12], and transient radiation fields may develop power-law tails at late times [1]. These examples motivate a practical and conceptual question that we address here: given a prescribed classical envelope Γ(t), what is a compatible pure-loss quantum master equation that reproduces the coherent-state mean trajectory, and under what conditions does the resulting evolution remain of (time-local) Lindblad type?

We answer this question using a correspondence principle strategy tailored to bosonic modes. The starting point is that a coherent state |α〉 provides a convenient quantum representative for a classical complex amplitude in the coherent-state/pure-loss correspondence; we therefore represent a decaying classical mode by the coherent-state trajectory |αΓ(t)〉. By imposing that the density operator ρ(t)=|αΓ(t)〉〈αΓ(t)| is an exact solution of the quantum dynamics, within the restricted class of single-mode, zero-temperature, vacuum-loss dynamics, we obtain a time-local master equation whose rate is fixed directly by the chosen envelope Γ(t). We explicitly emphasize that this ansatz imposes that the state remains pure, selecting a zero-temperature (vacuum) reservoir model. A thermal environment with the same amplitude damping generates fluctuations, mapping pure coherent states to mixed states. The proposed master equation thus provides a compatible quantum model under explicit pure-loss assumptions.

Furthermore, a time-dependent rate γ(t)≥0 does not imply quantum non-Markovianity or system memory. Following the standard divisibility criterion [13,14], dynamics with γ(t)≥0 are completely positive (CP) divisible and represent time-inhomogeneous Markovian processes. True quantum non-Markovianity requires negative rates (γ(t)<0) or explicit environmental memory, which are beyond the simple pure-loss construction presented here.

To explicitly clarify what is novel relative to earlier derivations of quantum damping from coherent-state properties (e.g., Ref. [15]), we note that while Ref. [15] explores time-dependent damping parameters conceptually, our work provides an explicit dictionary linking standard classical envelope solutions Γ(t) (spanning Ohmic, diffusive, KWW, and QNM cases) directly to time-local quantum generators, while explicitly addressing CP-divisibility criteria and the physical limits of macroscopic field quantization.

The manuscript is organized as follows. Section 2 summarizes representative classical decay regimes and corrected field profiles. Section 3 derives the associated time-local master equation, specifies admissibility conditions on Γ(t), gives explicit quantum channels, proves uniqueness, and clarifies CP-divisibility. Section 4 discusses the scope, environmental noise assumptions, macroscopic QED requirements, and comparisons with existing correspondence frameworks. Section 5 presents the conclusions.

## 2. Classical Decay Laws of Electromagnetic Fields

The temporal evolution of electromagnetic fields is governed by Maxwell’s equations [1,3]. The functional form of any observed decay, however, is not universal; it depends on the constitutive response of the medium, the relevant length and time scales, boundary conditions, and geometry [1,2]. For the correspondence approach pursued in the next section, the key classical object is an effective amplitude envelope Γ(t) that captures how the complex field amplitude of a given mode decreases in time. Throughout this manuscript, Γ(t) is defined strictly as the real dimensionless amplitude envelope (with rate γ/2) rather than the energy envelope (with rate γ). Specifically, for exponential decay with relaxation time τ, γ=2/τ. The purpose of this section is therefore not to provide an exhaustive review, but to identify representative decay laws and express them in a form that can be summarized by a mode envelope Γ(t).

### 2.1. Exponential Decay: Conductive Relaxation

A paradigmatic example is Ohmic relaxation in a homogeneous conductor. For finite conductivity σ and permittivity ε, free charge relaxes exponentially and the associated fields decay on the time scale τ:=ε/σ [1,2,3]:

(1) $$E\left(t\right)=E\left(0\right)\;e^{-t/\tau },\;\tau :=\frac{\varepsilon }{\sigma }.$$

In terms of our envelope language, a single-mode amplitude may be idealized as Γ(t)=exp(−t/τ), or, in quantum-optics convention for energy damping, Γ(t)=exp(−γt/2) with γ=2/τ. Exponential decay also arises as a good approximation for a long-lived mode of a high-*Q* resonator, where *Q* is the quality factor, when radiation leakage can be characterized by a single rate [11].

### 2.2. Diffusive Decay: Magnetic Field Penetration

When magnetic fields penetrate a conductor, the relevant dynamics is often diffusive. For an initially field-free conducting half-space (x>0) subjected to a maintained boundary field $B\left(0,t\right)=B_{0}$ for t>0, the solution to $\partial_{t}B=D\partial_{x}^{2}B$ (where $D={\left(\mu \sigma \right)}^{-1}$) is given by the complementary error function profile [1,2]:

(2) $$B\left(x,t\right)=B_{0}\;\mathrm{erfc}\left(\frac{x}{2\sqrt{Dt}}\right).$$

The boundary maximum remains fixed at $B_{0}$. On the other hand, an instantaneous pulse/delta-function initial field profile yields the Green’s function decay $B\left(x,t\right)∼t^{-1/2}e^{-x^{2}/\left(4Dt\right)}$. To use an algebraic power law as a dimensionless envelope Γ(t), one must introduce a characteristic reference time scale $t_{0}>0$ and a normalization factor, yielding $\Gamma \left(t\right)={\left(1+t/t_{0}\right)}^{-1/2}$ or ${\left(t/t_{0}\right)}^{-1/2}$ for $t\geq t_{0}$.

### 2.3. Oscillatory Damped Decay: Lossy Dielectrics and Conductors

Lossy media generically support damped waves with an oscillatory carrier and a decaying envelope. For a monochromatic plane wave the field can be written as [1]

(3) $$E\left(r,t\right)=E_{0}\;e^{-\kappa z}\cos\left(kz-\omega t\right),$$

where κ is the attenuation coefficient. To relate spatial attenuation $e^{-\kappa z}$ to an effective temporal mode envelope Γ(t), we consider a propagating wavepacket trajectory $z=v_{g}t$ (where $v_{g}$ is the group velocity), yielding an effective temporal attenuation factor $e^{-\kappa v_{g}t}$ that maps onto $\Gamma \left(t\right)=e^{-\gamma t/2}$ with $\gamma =2\kappa v_{g}$. Alternatively, in cavity configurations, E(r,t) represents a standing mode amplitude evaluated along a fixed position where decay is purely temporal. Treating an oscillatory envelope $\Gamma \left(t\right)=e^{-\gamma t/2}\cos\Omega t$ directly as a single real attenuation envelope causes sign changes and zero-crossings that introduce singularities into the master equation, as discussed in Section 3. Specifically, an envelope with a carrier cosΩt gives γ(t)=γ+2Ωtan(Ωt), that diverges at Ωt=π/2 and becomes negative on part of each cycle. The correct quantum description represents the field quadrature via the complex trajectory $\alpha \left(t\right)=\alpha e^{-i\Omega t-\gamma t/2}$ under the harmonic Hamiltonian $H_{0}=\hbar \Omega a^{†}a$. If one insists on treating an oscillatory envelope directly within a real time-dependent rate framework, the instantaneous rate γ(t) possesses periodic poles and negative intervals, meaning the claim of an instantaneous Lindblad form cannot apply throughout the entire evolution. Positivity of the instantaneous rate γ(t)≥0 (required for CP-divisibility) must be strictly distinguished from complete positivity of the integrated overall map $\Phi_{t,0}$.

### 2.4. Power-Law Decay: Radiation from Transient Sources

Transient sources can generate late-time radiation tails that decay algebraically. We note that a localized, smooth, compact-duration dipole pulse in vacuum generates a radiation field $E\left(t\right)\propto \ddot{d}\left(t-r/c\right)$, which vanishes identically after the pulse passes (in accordance with Huygens’ principle in 3 + 1 dimensional free space). Algebraic tails of the form E(t)∼1/t or $t^{-p}$ require specific physical mechanisms, such as propagation in dispersive media, backscattering off spatial inhomogeneities, or coupling to continuous spectral cutoffs/photonic band edges [16,17,18]. When such mechanisms yield a late-time decay, an admissible dimensionless envelope requires a regularization scale $t_{0}$, e.g., $\Gamma \left(t\right)={\left(1+t/\tau_{0}\right)}^{-p}$ for p>0.

### 2.5. Stretched Exponential: Disordered and Complex Media

In many disordered materials, glasses, polymers, porous media, and biological tissues, relaxation is broadly distributed in time and is not well described by a single Debye relaxation time. We emphasize that the Kohlrausch–Williams–Watts (KWW) function represents a constitutive material response or dielectric susceptibility relaxation, rather than a directly quantized free-field amplitude mode. A common empirical description is the KWW susceptibility [9,10,19,20]

(4) $$\chi \left(t\right)\propto e^{-{\left(t/\tau \right)}^{\beta }},\;0<\beta <1,$$

that interpolates between an exponential (β=1) and a slow, broad relaxation (β<1). For a step field that is switched off at t=0, the relaxation function Φ(t) governs the subsequent decay of the polarization. For a step field that is switched on at t=0, the corresponding response is proportional to 1−Φ(t). The susceptibility response kernel is

(5) $$\chi \left(t\right)=-\frac{d\Phi (t)}{dt},$$

that describes the linear response to arbitrary field histories. The field or polarization can be modeled with an envelope of the form $\Gamma \left(t\right)=\exp[-{\left(t/\tau \right)}^{\beta }/2]$ [9,21]. For 0<β<1, Γ(t) is continuous for t≥0 but non-differentiable at t=0. While its instantaneous rate γ(t) diverges as $t\to 0^{+}$, this singularity is integrable ($\int_{0}^{t}\gamma \left(s\right)ds<\infty$), allowing a well-defined continuous quantum channel for all t≥0.

### 2.6. Multi-Mode Exponential Sum: Quasi-Normal Modes

Open systems, leaky cavities, antennas, and plasmonic resonators are often inherently multi-modal. In that setting, the field can be expanded in quasi-normal modes (QNMs) with complex eigenfrequencies ${\tilde{\omega }}_{n}:=\omega_{n}-i\gamma_{n}/2$ [11,12,22]:

(6) $$E\left(t\right)=\sum_{n}c_{n}\;e^{-i{\tilde{\omega }}_{n}t}=\sum_{n}c_{n}\;e^{-\gamma_{n}t/2}\;e^{-i\omega_{n}t}.$$

Equation (6) represents a sum of distinct spatial and spectral modes, rather than a single canonical quantum mode. Assuming independent canonical modes and reservoirs, the total multi-mode evolution is governed by a sum of independent dissipators. Distinct, generally nonorthogonal QNMs in open cavities do not by themselves justify an exact sum of independent dissipators without accounting for modal cross-coupling and excess noise. Furthermore, QNMs in open non-Hermitian systems are nonorthogonal and subject to Petermann excess noise [23], requiring specialized multi-mode master equations.

## 3. Correspondence Principle and the Quantum Master Equation

In this section we turn the classical envelope Γ(t) into a quantum dynamical law for the density operator [5,6,14]. The restricted goal here is to construct a single-mode, time-local vacuum-loss master equation whose coherent-state solution reproduces the correspondence ansatz ρ(t)=|αΓ(t)〉〈αΓ(t)| (that acts as the defining input assumption rather than an emergent property) for an initially coherent field [15,24,25,26], and to express the resulting dissipative rate directly in terms of Γ(t).

To ensure mathematical rigor and physical validity, we state the precise admissibility conditions for the envelope Γ(t):

1. Normalization and Positivity: Γ(0)=1 and 0<Γ(t)≤1 for all t≥0.
2. Monotonicity: $\dot{\Gamma }\left(t\right)\leq 0$ for all t≥0, ensuring γ(t)≥0.
3. Differentiability: Γ(t) is continuously differentiable for t>0 (or has integrable derivative singularities at t=0, as in the stretched exponential case).

If Γ(t) vanishes or changes sign at some $t_{0}$, Equation (14) becomes singular ($\gamma (t_{0})\to \infty$). At a zero-crossing, the map sends every initial state to the vacuum |0〉〈0|. A subsequent revival of amplitude cannot be generated by a time-local propagator acting solely on the vacuum state without external driving or explicit bath memory. We note that the requirement Γ(t)≤1 is mandatory for complete positivity without added noise, and any growing envelope Γ(t)>1 represents amplification, which cannot be completely positive without introducing noise/diffusion terms.

At the classical level, a single field mode is characterized by a complex amplitude α(t)=αΓ(t). With this parametrization, phase rotations reside in the complex phase of α, while Γ(t) controls the damping entering the time-local generator derived below.

### 3.1. Derivation

Within the assumptions of Section 3, our goal is to determine the time-local generator $L_{t}$ such that $\dot{\rho }\left(t\right)=L_{t}\left[\rho \left(t\right)\right]$ reproduces the correspondence trajectory ρ(t)=|αΓ(t)〉〈αΓ(t)|. We will show that $L_{t}$ can be cast in GKSL form with a single jump operator *a*, i.e., $L_{t}\left[\rho \right]=\gamma \left(t\right)\;D\left[a\right]\rho$, where $D\left[a\right]\rho :=a\rho a^{†}-\frac{1}{2}\left\{a^{†}a,\rho \right\}$. We proceed constructively by computing the first-order increment ρ(t+h)−ρ(t) and then taking the limit h→0.

We start from the coherent-state expansion in the Fock basis,

(7) $$|\alpha \;\Gamma \left(t\right)\rangle =e^{-{\left|\alpha \right|}^{2}\Gamma^{2}\left(t\right)/2}\sum_{n=0}^{\infty }\frac{{\left[\alpha \Gamma \left(t\right)\right]}^{n}}{\sqrt{n!}}\;|n\rangle ,$$

which yields the density operator

(8) $$\rho \left(t\right)=e^{-{\left|\alpha \right|}^{2}\Gamma^{2}\left(t\right)}\sum_{n,m=0}^{\infty }|n\rangle \langle m|\;\frac{\alpha^{n}\alpha^{*m}\;\Gamma^{n+m}\left(t\right)}{\sqrt{n!\;m!}}.$$

To compute ρ(t+h) to first order in *h*, we expand each factor that depends on *t*. Using $\Gamma \left(t+h\right)=\Gamma \left(t\right)+h\dot{\Gamma }\left(t\right)+O\left(h^{2}\right)$ and retaining only O(h) terms gives the set of expansions

(9a) $$\begin{array}{cc} \Gamma (t+h) & \approx \Gamma \left(t\right)+h\;\dot{\Gamma }\left(t\right), \end{array}$$

(9b) $$\begin{array}{cc} \;\Gamma^{n+m}\left(t+h\right) & \approx \Gamma^{n+m}\left(t\right)+\left(n+m\right)h\;\Gamma^{n+m}\left(t\right)\;\frac{\dot{\Gamma }\left(t\right)}{\Gamma (t)},\\ e^{-{\left|\alpha \right|}^{2}\Gamma^{2}\left(t+h\right)} & \approx e^{-{\left|\alpha \right|}^{2}\Gamma^{2}\left(t\right)}\left[1-{2h|\alpha |}^{2}\Gamma \left(t\right)\dot{\Gamma }\left(t\right)\right] \end{array}$$

(9c) $$\begin{array}{cc} \; & =e^{-{\left|\alpha \right|}^{2}\Gamma^{2}\left(t\right)}\left[1-{2h|\alpha |}^{2}\Gamma^{2}\left(t\right)\;\frac{\dot{\Gamma }\left(t\right)}{\Gamma (t)}\right]. \end{array}$$

Substituting Equations (9a)–(9c) into Equation (8) and collecting all O(h) contributions yields

(10) $$\begin{array}{c} \rho \left(t+h\right)\approx e^{-{\left|\alpha \right|}^{2}\Gamma^{2}\left(t\right)}\left[1-{2h|\alpha |}^{2}\Gamma^{2}\left(t\right)\;\frac{\dot{\Gamma }\left(t\right)}{\Gamma (t)}\right]\\ \;\times \sum_{n,m}\;|n\rangle \langle m|\;\frac{\alpha^{n}\alpha^{*m}\Gamma^{n+m}\left(t\right)}{\sqrt{n!\;m!}}\left[1+h\left(n+m\right)\frac{\dot{\Gamma }\left(t\right)}{\Gamma (t)}\right]. \end{array}$$

The expression (10) is still written componentwise in the Fock basis. To identify a compact operator form, we relate the factors *n* and *m* in the sum to number-operator actions. Using $a|n\rangle =\sqrt{n}\;|n-1\rangle$ and $a^{†}|n\rangle =\sqrt{n+1}\;|n+1\rangle$, one finds the operator identities

(11a) $$\begin{array}{cc} a\rho \left(t\right)a^{†} & ={\left|\alpha \right|}^{2}\Gamma^{2}\left(t\right)\;\rho \left(t\right), \end{array}$$

(11b) $$\begin{array}{cc} \;a^{†}a\;\rho \left(t\right) & =e^{-{\left|\alpha \right|}^{2}\Gamma^{2}\left(t\right)}\sum_{n,m}n\;|n\rangle \langle m|\;\frac{\alpha^{n}\alpha^{*m}\Gamma^{n+m}\left(t\right)}{\sqrt{n!\;m!}}, \end{array}$$

(11c) $$\begin{array}{cc} \;\rho \left(t\right)\;a^{†}a & =e^{-{\left|\alpha \right|}^{2}\Gamma^{2}\left(t\right)}\sum_{n,m}m\;|n\rangle \langle m|\;\frac{\alpha^{n}\alpha^{*m}\Gamma^{n+m}\left(t\right)}{\sqrt{n!\;m!}}. \end{array}$$

In particular, Equations (11b) and (11c) show that the combination (n+m) appearing in Equation (10) corresponds to $a^{†}a\;\rho +\rho \;a^{†}a$, while Equation (11a) accounts for the term proportional to ${\left|\alpha \right|}^{2}\Gamma^{2}\left(t\right)$.

Using these identities and expanding Equation (10) to first order, we obtain the finite-difference form

(12) $$\rho \left(t+h\right)\approx \rho \left(t\right)+h\;\frac{\dot{\Gamma }\left(t\right)}{\Gamma (t)}\left[a^{†}a\;\rho \left(t\right)+\rho \left(t\right)\;a^{†}a-2a\rho \left(t\right)a^{†}\right].$$

Dividing by *h* and taking h→0 gives the master equation

(13) $$\dot{\rho }=\frac{\dot{\Gamma }\left(t\right)}{\Gamma (t)}\left(a^{†}a\;\rho +\rho \;a^{†}a-2a\rho a^{†}\right).$$

This expression can be rewritten in the standard dissipator form

(14) $$\dot{\rho }=\gamma \left(t\right)\left(a\rho a^{†}-\frac{1}{2}\left\{a^{†}a,\rho \right\}\right),\;\gamma \left(t\right):=-2\;\frac{\dot{\Gamma }\left(t\right)}{\Gamma (t)}.$$

Here {A,B}:=AB+BA denotes the anticommutator. The right-hand side of Equation (14) has the standard Gorini–Kossakowski–Sudarshan–Lindblad (GKSL) structure [27,28]. The present construction follows a philosophy similar to that used in invariant-based treatments of the time-dependent quantum harmonic oscillator, where classical dynamical quantities are elevated to quantum operators while preserving the corresponding classical limit. In particular, Fernández Guasti and Moya-Cessa showed how the Ermakov–Lewis invariant may be reformulated in terms of amplitude and phase variables that directly correspond to their classical counterparts [29].

### 3.2. Sufficient Conditions and Generator Consistency

Rather than proving microscopic necessity, we establish Equation (14) as a consistent sufficient model: under the assumption of a linear, rotating-wave zero-temperature microscopic interaction, the generator produces the prescribed pure-loss dynamics for arbitrary initial states. Linearity of the chosen generator and the CPTP channel establish its action on arbitrary states; they do not uniquely identify a single microscopic realization, as alternative environmental configurations (such as nonzero temperature or additional phase shifts) can yield identical mean trajectories with different state evolution. A passive linear vacuum-bath model supplies a realization under suitable initial-factorization assumptions, but necessity has not been proved. In a general setting with complex response G(t), the master equation for a mode linearly coupled to a zero-temperature bosonic bath [30] takes the form $\dot{\rho }=-iS\left(t\right)\left[a^{†}a,\rho \right]+\gamma \left(t\right)D\left[a\right]\rho$, with γ(t)=−2Re(G˙/G) and S(t)=−Im(G˙/G). Restricted to real Γ(t), S(t)=0 identically.

### 3.3. Quantum Channel Representation and CP-Divisibility

For any admissible envelope Γ(t), the integrated dynamical map $\Phi_{t,0}$ from t=0 to *t* is a completely positive, trace-preserving (CPTP) quantum channel. In Kraus representation, the evolution of an arbitrary initial state ρ(0) is given by

(15) $$\rho \left(t\right)=\Phi_{t,0}\left[\rho \left(0\right)\right]=\sum_{k=0}^{\infty }A_{k}\left(t\right)\rho \left(0\right)A_{k}^{†}\left(t\right),\;A_{k}\left(t\right)=\sqrt{\frac{{\left[1-\eta \left(t\right)\right]}^{k}}{k!}}\eta {\left(t\right)}^{\frac{a^{†}a}{2}}a^{k},$$

where $\eta \left(t\right)=\Gamma^{2}\left(t\right)$ represents the field energy transmissivity (0≤η(t)≤1). For intermediate propagation between $t_{1}$ and $t_{2}$ ($t_{2}\geq t_{1}\geq 0$), the map decomposes as $\Phi_{t_{2},0}=\Phi_{t_{2},t_{1}}\circ \Phi_{t_{1},0}$. The intermediate propagator $\Phi_{t_{2},t_{1}}$ is itself a valid CPTP channel with effective transmissivity

(16) $$\eta \left(t_{2},t_{1}\right)={\left[\frac{\Gamma (t_{2})}{\Gamma (t_{1})}\right]}^{2}\leq 1.$$

Because $\eta (t_{2},t_{1})\leq 1$ holds for all $t_{2}\geq t_{1}$ whenever Γ(t) is monotonically decaying, the dynamic is CP-divisible. Under the divisibility criterion [13,14], a time-dependent rate γ(t)≥0 generates a *time-inhomogeneous Markovian process*. Explicit time dependence of γ(t) does not constitute quantum non-Markovianity or memory dependence, as the rate at time *t* depends only on the current time parameter *t*, not on previous states of the system. Before claiming non-Markovian behavior, one must adopt a formal definition—such as CP-divisibility or non-monotonicity of information flow—and demonstrate actual memory effects (e.g., rate positivity violations γ(t)<0 or backflow of trace distance), that are absent in this CP-divisible setup.

We clarify that if Γ(t) were non-monotonic, γ(t)<0 at intervals where Γ(t) increases. During those intervals, the time-local equation is not of Lindblad form. However, as long as 0<Γ(t)≤1, the overall map $\Phi_{t,0}$ remains CP, but it loses CP-divisibility. For non-monotonic Γ(t), information flows back from the environment; for two coherent states $|\alpha_{1}\rangle$ and $|\alpha_{2}\rangle$, the trace distance $D\left(t\right)=\sqrt{1-\exp(-\Gamma^{2}\left(t\right)|\alpha_{1}-\alpha_{2}{|}^{2})}$ increases whenever Γ(t) increases [14].

### 3.4. Properties and Consistency Checks

The master Equation (14) was engineered to reproduce the coherent-state trajectory |αΓ(t)〉; nevertheless, it is useful to make explicit a few structural properties that any physically meaningful dissipative evolution should satisfy. First, Equation (13) is trace preserving: taking the trace and using cyclicity, $\mathrm{Tr}\left(a\rho a^{†}\right)=\mathrm{Tr}\left(a^{†}a\rho \right)$, yields $\mathrm{Tr}\dot{\rho }=0$, so Trρ(t) is conserved. Crucially, because Equation (14) is a linear generator, Equation (14) governs the evolution of any initial state ρ(0) and it holds for all states (Fock states, squeezed states, thermal states, etc.) under the stated sufficient-model assumptions.

For the mean photon number $\bar{n}\left(t\right)=\mathrm{Tr}\left(a^{†}a\rho \left(t\right)\right)={\left|\alpha \right|}^{2}\Gamma^{2}\left(t\right)$, differentiation yields

(17) $$\frac{d\bar{n}}{dt}=2{\left|\alpha \right|}^{2}\Gamma \left(t\right)\dot{\Gamma }\left(t\right)=-\gamma \left(t\right)\bar{n}\left(t\right).$$

This rate equation correctly captures the energy damping of the mode.

### 3.5. Examples

The correspondence rule provides a direct recipe: once a classical envelope Γ(t) is specified, the quantum dynamics follows from $\gamma \left(t\right)=-2\dot{\Gamma }\left(t\right)/\Gamma \left(t\right)$. For γ(t)≥0, Equation (14) generates a bosonic pure-loss (amplitude-damping) channel with transmissivity $\eta \left(t\right)=\Gamma^{2}\left(t\right)$ [5,6].

#### 3.5.1. Exponential Decay

For Γ(t)=exp(−γt/2) one has $\dot{\Gamma }/\Gamma =-\gamma /2$ and therefore γ(t)=γ (constant). Equation (14) reduces to the standard optical master equation,

(18) $$\dot{\rho }=\gamma \left(a\rho a^{†}-\frac{1}{2}a^{†}a\rho -\frac{1}{2}\rho a^{†}a\right),$$

that is time-homogeneous and defines a dynamical semigroup [27,28].

#### 3.5.2. Power-Law Decay

For a regularized algebraic envelope $\Gamma \left(t\right)={\left(1+t/\tau_{0}\right)}^{-p}$ with p>0, the instantaneous damping rate becomes

(19) $$\gamma \left(t\right)=\frac{2p}{\tau_{0}+t},$$

which decreases monotonically in time. Since γ(t)>0 for all t≥0, the channel remains CP-divisible and corresponds to time-inhomogeneous Markovian dynamics.

#### 3.5.3. Stretched Exponential

For the stretched exponential envelope $\Gamma \left(t\right)=\exp[-{\left(t/\tau \right)}^{\beta }/2]$ with 0<β<1, one finds

(20) $$\gamma \left(t\right)=\frac{\beta }{\tau }{\left(\frac{t}{\tau }\right)}^{\beta -1}.$$

Although γ(t) diverges at t=0, its integral $\int_{0}^{t}\gamma \left(s\right)ds={\left(t/\tau \right)}^{\beta }$ is finite and well-defined. Thus, a continuous CP-divisible quantum channel is defined for all t≥0.

## 4. Discussion

The time-dependent decay laws of electromagnetic fields are not universal; rather, they emerge from Maxwell’s equations under specific physical conditions. The central point of this work is that, once such a classical decay law is distilled into an effective envelope Γ(t) for a mode amplitude, it can be promoted to a quantum description by the correspondence rule

(21) $$\gamma \left(t\right):=-2\;\frac{\dot{\Gamma }\left(t\right)}{\Gamma (t)}.$$

The mapping developed here complements earlier correspondence principle constructions for closed time-dependent oscillators. Whereas Fernández Guasti and Moya-Cessa [29] related classical amplitude-phase invariants to quantum invariant operators of the time-dependent harmonic oscillator, the present work associates classical decay envelopes with quantum dissipative generators governing bosonic pure-loss dynamics.

### Physical Scope, Environmental Assumptions, and Comparison

It is essential to clarify what physical assumptions underpin this construction. The requirement that an initial pure coherent state |α〉 remains pure |αΓ(t)〉 uniquely selects a zero-temperature (vacuum) reservoir loss model. The master equation derived here is not a universal consequence of classical decay alone; it should be read as one compatible quantization rule, not as the unique open-system dynamics associated with the measured classical envelope but specifically describes vacuum-loss dynamics in highly isolated environments. By explicitly presenting this framework as a compatible quantum model under pure-loss assumptions, we clarify that classical attenuation alone does not uniquely fix the noise environment.

Furthermore, integrating Equation (14) demonstrates that the constructed dynamics is formally equivalent to standard quantum vacuum loss reparametrized by an effective accumulated time scale $\tau \left(t\right)=\int_{0}^{t}\gamma \left(s\right)ds=-2\ln\Gamma \left(t\right)$. Regarding microscopic foundations, atomic pseudomode treatments (such as Ref. [30]) establish oscillator damping formally via modal decompositions, whereas standard atomic references (e.g., Ref. [14]) treat two-state atomic populations; our bosonic treatment is derived directly for field modes while utilizing atomic pseudomode analogies for multi-rate environments. The utility of the present mapping lies in providing a direct dictionary linking classical boundary/medium decay laws to time-local quantum generators.

We also emphasize that for classical processes such as charge relaxation, magnetic diffusion, or KWW polarization relaxation, the physical system is overdamped and non-propagating; strictly speaking, there is no underlying oscillator with $H=\hbar \omega a^{†}a$ or photon excitation. Applying a coherent-state master equation to such systems must be understood as a formal map for an effective bosonic degree of freedom. Moreover, quantizing electromagnetic fields in complex, dispersive, or absorbing media rigorously requires macroscopic QED (e.g., Huttner and Barnett [31], Gruner and Welsch [32]), where field operators are expressed via continuous Langevin noise operators in the dielectric.

Table 1 collects representative examples and illustrates how they should be read.

In Table 1, the diffusive peak damping rate is corrected to $1/(\tau_{0}+t)$ for $\Gamma \left(t\right)={\left(1+t/\tau_{0}\right)}^{-1/2}$, eliminating the factor-of-two discrepancy with $\Gamma \left(t\right)∼t^{-1/2}$. For multi-mode QNM expansions, single-mode effective generators are approximations; an exact quantum treatment requires independent jump operators $a_{n}$ with rates $\gamma_{n}$ for each distinct physical mode.

## 5. Conclusions

We have presented a correspondence rule that promotes prescribed classical amplitude envelopes to single-mode, zero-temperature vacuum-loss dynamics. Starting from a classical envelope Γ(t) for the complex field amplitude, we derived a time-local master equation whose generator is fixed by the logarithmic derivative $\gamma \left(t\right)=-2\dot{\Gamma }\left(t\right)/\Gamma \left(t\right)$, yielding Equation (14).

We have clarified that the correspondence ansatz |αΓ(t)〉 specifically selects a zero-temperature vacuum reservoir model, as thermal environments introduce state mixing and fluctuations not captured by classical first moments alone. We explicitly formulate the model as a compatible quantum description under defined pure-loss constraints, acknowledging that classical damping alone does not constrain environmental noise. Furthermore, time dependence in the damping rate γ(t)≥0 corresponds to time-inhomogeneous Markovian dynamics under CP-divisibility criteria, rather than quantum memory effects. By distinguishing overall map complete positivity from instantaneous rate positivity, we address the limits and singularities associated with oscillating envelopes. The presented corrections to classical inputs—including error function diffusion boundary conditions, carrier separation in oscillating fields, and proper regularization of algebraic tails—provide a foundation for mapping classical electrodynamic decay laws onto quantum master equations.

## Figures and Tables

**Table 1:** Classical decay regimes and the corresponding quantum master equation.

Regime	Classical Envelope Γ(t)	Quantum Rate γ(t)
Exponential	exp(−γt/2)	γ (constant)
Diffusive (peak decay)	${\left(1+t/\tau_{0}\right)}^{-1/2}$ (regularized)	$1/(\tau_{0}+t)$ (rate corrected)
Damped oscillation	exp(−γt/2) (rotating frame)	γ (envelope rate)
Power law	${\left(1+t/\tau_{0}\right)}^{-p}$	$2p/(\tau_{0}+t)$
Stretched exponential	$\exp[-{\left(t/\tau \right)}^{\beta }/2]$	$\left(\beta /\tau \right){\left(t/\tau \right)}^{\beta -1}$ (t>0)
Multi-mode QNM	Mode-dependent sum	Multi-channel dissipators

## Data Availability

The data presented in this study are available on request from the corresponding author.
